# Species-specific AAVR dominates species-tropism of adeno-associated virus (AAV) vectors

**DOI:** 10.21203/rs.3.rs-8198480/v1

**Published:** 2025-12-05

**Authors:** Mark Kay, Yuqian Jiang, Yiming Liu, Yuqing Jing, Feijie Zhang, Angela Lin

**Affiliations:** Stanford University; Stanford University; Stanford University; Stanford University; stanford; Stanford University

**Keywords:** Adeno Associated Virus (AAV), AAVR, species tropism, AAV-LK03, gene therapy

## Abstract

The variation in AAV vector mediated transduction between species makes it difficult to predict and translate from preclinical animal studies to clinical trials. We previously demonstrated that a 265G insertion (AAV-AM) into the AAV-LK03 primate selective capsid allows robust transduction between species. Here we demonstrate that the capsid species specificity cannot be complemented in trans and hence the transduction properties are cis mediated. We found that the 265G is in a surface-exposed region of AAV virion, facilitating its accessibility for binding to the universal AAVR receptor. We further demonstrated that human AAVR (hAAVR) supplementation rescued the low murine transduction of AAV-LK03 in vitro and in vivo. Sequence swap experiments demonstrated the four amino acid variation of mouse vs human PKD2 domain was important for the varied species tropism of AAV-LK03, with I426V having the greatest effect. Our findings imply that transduction efficiencies between various AAV capsids are substantially influenced by sequence variation in the AAVR protein observed between species. This may facilitate better approaches for translating preclinical to clinical application of AAV-based gene therapeutics.

## Introduction

Despite its tremendous promise, the variance in species tropism is an inherent characteristic of AAV vectors that prolongs the time frame required for translating preclinical models to clinical trials^1^.

For example, AAV-LK03, which was selected from a shuffled capsid library and selected from a humanized liver mouse model^2^, provided high rates of transduction in primates^3,4^ and ultimately in a hemophilia A clinical trial^5^, does not transduce murine liver or cells well^6^. Later we demonstrated that the differences in AAV-LK03 transduction between species was related to differences in epigenome formation between species. And by a single 265G insertion into the LK03 capsid (AAV-AM), we restored transduction in murine systems^6^.

To further unravel the mechanisms involved, we demonstrate that the species tropism occurs *in cis* and was not impacted by either transfection of capsid proteins or co-transduction with better-transducing AAVs. We also found that 265G locates in a surface-exposed region of an AAV virion, facilitating its accessibility for binding with other factors. Furthermore, we demonstrated that the species origin of AAVR (*KIAA0319L*) has a dramatic effect on the varied transduction observed between species. In this study, we explored the AAVR regions that varied by species and how these non-conserved sequences play an important role in species-selective AAV transduction. Our findings provide insights into species tropism of AAV vectors and contribute to translation of AAV-mediated therapies from preclinical characterization to clinical trials, facilitating gene therapies of safety and reliability.

## RESULTS

### Species tropism occurs in cis

**Figure 1.**
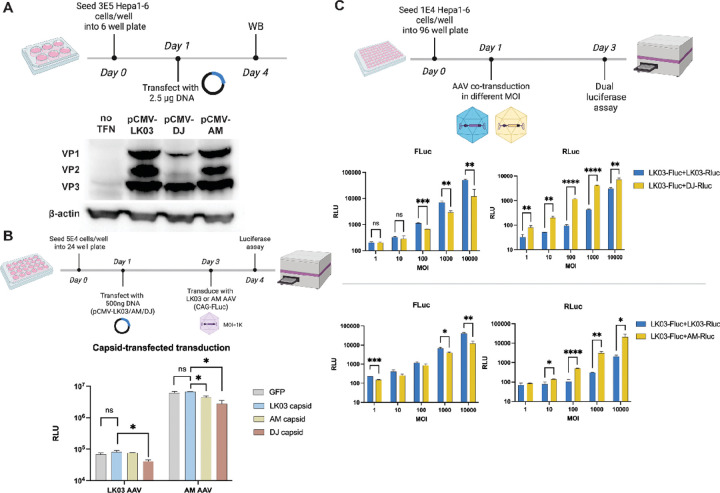
Species Tropism of AAV-LK03 occurs *in cis*. (A) Western blotting validating the expression of VP proteins in Hepa1–6 cells transfected with plasmids encoding LK03/DJ/AM capsids. (B) Luciferase activities were measured from AAV-LK03 or AAV-AM transduced Hepa1–6 cells transfected with one of three plasmids expressing the indicated capsid protein. (C) Dual-luciferase activities were measured from co-transduction with two AAV-vectors, AAV-LK03 expressing Firefly luciferase and a second AAV either LK03, DJ or AM expressing Renilla luciferase.

To establish if the species selectivity could be affected by another capsid in *trans*, we transfected murine Hepa1–6 cells with plasmids expressing capsid proteins from AAV-DJ, AM or AAV-LK03 (Fig. 1A–B). Each showed three clear bands representing VP1/2/3 capsid proteins by western blotting (Fig. 1A). We then tested if the production of exogenous capsid proteins affected AAV transduction. As shown in Fig. 1B, exogenous expression of capsid proteins from AAV-AM or DJ in recipient cells failed to boost AAV-LK03 transduction. We then tested if co-transduction of AAV-AM or DJ with AAV-LK03 would enhance transduction in mouse cells (Fig. 1C). We used a dual luciferase assay to distinguish the firefly luciferase (FLuc) expressed from AAV-LK03 from Renilla luciferase (RLuc) expressed from an AAV-AM or DJ vector. The results showed that co-transduction was unable to enhance the low transduction of AAV-LK03 in Hepa1–6 cells. Together the results from these two experiments, strongly support that the species selectivity of AAV-LK03 cannot be complemented by more robust rodent transducing capsids.

### AAVR is a host factor contributing to species tropism

**Figure 2.**
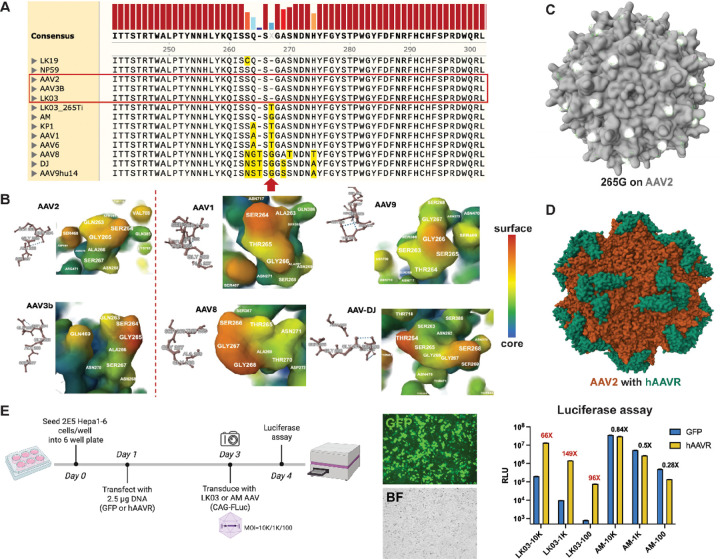
AAVR is a host factor responsible for species tropism. (A) Alignment of capsid sequences of multiple AAV serotypes. As gated in red, AAV2, AAV3B and AAV-LK03 shared the same sequence in this region. As highlighted in yellow, site 265 varies across AAV serotypes. (B) Cryo-EM structures of representative AAVs from Protein Data Bank, focusing on site 265 and colored based on surface accessibility. Red and orange indicate the most surface-exposed residues. PDB ID: 6IH9 (AAV2); 8A9U (AAV3b); 6JCR (AAV1); 6PWA (AAV8); 7WJW (AAV9); 3J1Q (AAV-DJ). Genome-containing AAV2 virion structure was shown in (C) (PDB ID: 6IH9) and AAV2 bound with hAAVR was shown in (D) (PDB ID: 6IHB). (E) Hepa1–6 cells were transfected with GFP or hAAVR expression plasmids and transduced with AAV-LK03 or AM. Luciferase activity was then measured. Brightfield (BF) and GFP images indicating the high transfection efficiency.

Given a single 265G insertion in LK03 capsid rescued the species selective tropism^6^, we sought to determine the localization and potential structural changes made within the virion. As shown in Fig. 2A, alignment of capsid sequences from multiple AAV serotypes revealed that the region surrounding the 265G is relatively conserved among serotypes. In addition, AAV-LK03 shares the same sequence as its parental serotype AAV2 and AAV3b in this region. To visualize this site, we examined surface exposed regions based on the cryo-EM structures of AAV2, AAV3b, AAV1, AAV8, AAV9 and AAV-DJ, as depicted in colored regions in Fig. 2B. The 265G was consistently located in a surface-exposed region for these AAV serotypes. Moreover, as shown in Fig. 2C, 265G sites from all the sixty VP monomers of AAV2 particle are surface-exposed, allowing for potential binding with other host factors.

KIAA0319L or AAVR was identified as an essential host factor for AAV transduction previously by the Carette group^7^. Further structural studies revealed binding regions of AAV particles with PKD domains of AAVR^8^. Interestingly, 265G overlaps with the regions where AAVR binds directly with AAV capsids. Based on the Cryo-EM structure of AAV2 bound with AAVR, there are sixty PKD2 peptides interacting with an AAV2 virion (Fig. 2D). Inspired by this, we hypothesized that species-specific AAVR may play a role in determining species tropism of AAV vectors.

Overexpression of AAVR from the same species has been shown to increase AAV transduction in some cases^9^. We elected to investigate how supplementation of AAVR across different species might affect AAV transduction. Since AAV-LK03 showed poor transduction in murine cells *in vitro* and *in vivo*, we first investigated if overexpression of human AAVR (hAAVR) rescued low transduction of LK03 in mouse cells (Fig. 2E). Transfection of an hAAVR expression plasmid enhanced AAV-LK03 luciferase expression by 66–149 times reaching the same level of transduction using the 265G variant, AAV-AM. In comparison, addition of hAAVR did not further increase AAV-AM based-expression. In fact, AAV-AM transduction was slightly reduced with hAAVR transfection, which may have been due to competition and a slight preference for mouse AAVR (mAAVR). The exogenous reconstitution of hAAVR did not change AAV-LK03 or AAV-AM vector copy number (VCN) isolated from nuclear lysates (Figure S1A), suggesting that hAAVR-mediated boosting of the luciferase signals was not due to increased nuclear entry.

### hAAVR supplement enhances LK03 in multiple murine cell lines and is transgene independent.

**Figure 3.**
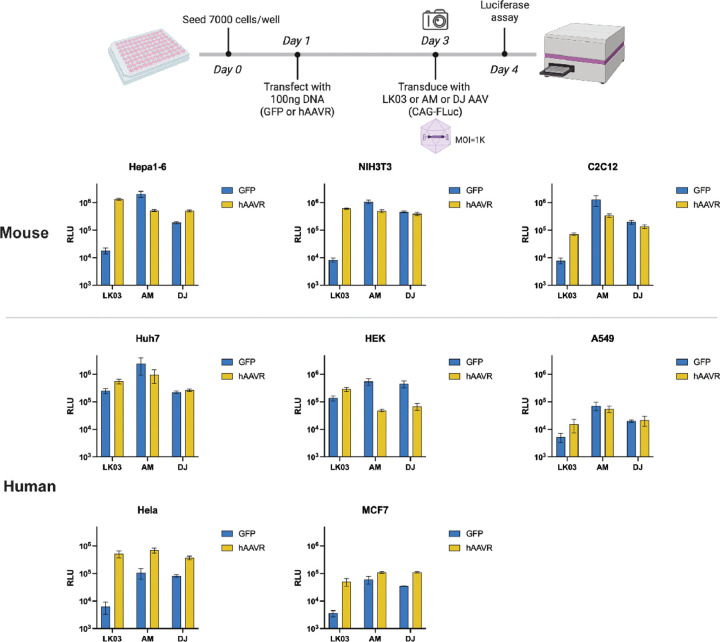
hAAVR supplementation enhances AAV-LK03 mediated transgene expression in multiple murine cell lines. Experimental scheme is shown on top. Luciferase activities were measured in multiple cell lines (murine: Hepa1–6, NIH3T3, C2C12; human: Huh7, HEK293, A549, Hela and MCF7) transduced with AAV-LK03/AM/DJ vector expressing FLuc. The cells were transfected with GFP (control) or hAAVR expression plasmids.

To determine the generality of hAAVR supplementation, we evaluated AAV-LK03 transduction with plasmid-transfected hAAVR supplementation in various murine and human cell lines (Fig. 3). The controls received a GFP expressing plasmid and the relative transfection efficiencies in the different cell lines were similar (Fig. S2A). Consistent with what we observed in the murine Hepa1–6 cell line, hAAVR overexpression in NIH3T3 and C2C12, murine fibroblast and myoblast cells, respectively also rescued the low transduction of AAV-LK03 boosting transgene expression to levels comparable to that achieved by AAV-AM and DJ. The results in the human cell lines were more varied. In Hela and MCF7 cells, hAAVR supplementation resulted in an almost 100x and 50x enhancement, respectively in AAV-LK03 transduction while there was little enhancement in Huh7, HEK293, and A549 cells. We also examined the expression level of endogenous AAVR in these cell lines (Figure S2B-C). Notably, HEK293 cells showed two-fold higher hAAVR expression than other human cell lines and in general hAAVR transcripts in human cell lines were 2–3 fold higher than mAAVR in mouse cell lines, revealed by previously published RNAseq data^10^ (Figure S2D, E). This suggests that there are additional factors affecting AAV transduction depending on cell types.

We confirmed the effect of hAAVR was not dependent on the expressed transgene by transducing murine Hepa1–6 cells with either AAV-LK03 or AAV-AM delivering a CAG-tdTomato expression cassette (Figure S3A-C). Cell imaging and results from flow cytometry showed that hAAVR supplement enhanced both the percentage of tdTomato positive cells and the mean fluorescence intensity (MFI) for AAV-LK03 transduction. As with the luciferase reporter, the reduction in AAV-AM with overexpressed hAAVR was also observed for the tdTomato reporter (Figure S3C). Taken together, the relative amount and type (hAAVR vs mAAVR) to reach maximal transduction varies between cell type and AAV capsid used.

### Endogenous mAAVR removal does not further enhance transduction with hAAVR supplement.

**Figure 4.**
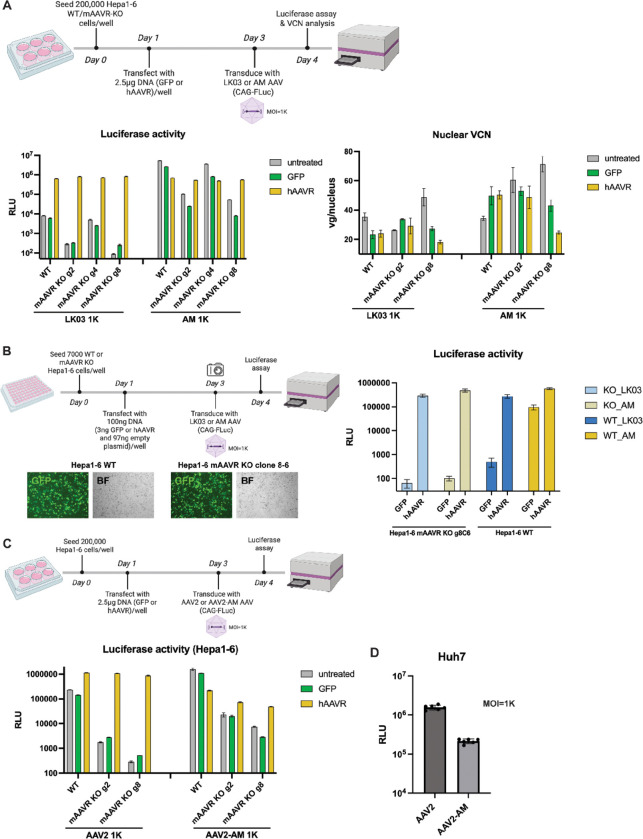
mAAVR knockout does not further enhance transduction with hAAVR supplementation. (A-C) The experimental design is shown. (A) Luciferase activities or nuclear VCN analysis in wildtype or mAAVR knockout Hepa1–6 cells transduced with AAV-LK03 or AM with plasmid-mediated GFP or hAAVR supplementation. (B) mAAVR knockout Hepa1–6 clone 8–6 cells or wildtype Hepa1–6 cells were transduced with AAV-LK03 or AAV-AM expressing the Fluc transgene, with prior transfection with plasmids expressing hAAVR or GFP. Cells were measured for luciferase activities. GFP images were taken on day 3 to indicate transfection efficiency. (C) Wildtype or mAAVR knockout Hepa1–6 cells were transfected with GFP or hAAVR expression plasmids and then transduced with AAV2 or AAV2-AM expressing the FLuc transgene. Cells were measured for luciferase activity. (D) Huh7 cells were transduced by AAV2 and AAV2-AM expressing the FLuc transgene and measured for luciferase activity.

To study the effect or hAAVR expression in the absence of endogenous mAAVR, we generated an mAAVR knockout Hepa1–6 cell line (Fig. 4 and S4). Results showed that the PKD2-targeting gRNA resulted in mAAVR depletion determined by the mRNA and protein levels (Figure S4B-D). Genotyping of clone 8 – 6 revealed a 2 bp deletion and a 35 bp insertion for the two alleles (Figure S4G).

We then seeded mAAVR knockout single cell clones and showed that they could not be transduced by rAAV vectors further establishing an mAAVR knockout phenotype (Figure S4E-F).

After plasmid-mediated reconstitution of the Hepa1–6 mAAVR knockout cells (both population cells and single cell clones), hAAVR overexpression restored AAV-LK03 and AAV-AM transduction to similar levels (Fig. 4A–B). However, in the absence of wildtype mAAVR, overexpression of hAAVR failed to further increase AAV-transduction to levels greater than that obtained in hAAVR transfected wildtype cells. These results established that the endogenous level of mAAVR does not restrict the upper limit of AAV transduction in the presence of high hAAVR expression.

Using mAAVR knockout Hepa1–6 cell lines, we tested AAV2 and AAV2-AM, the later of which harbors the 265G insertion in the AAV2 capsid in transduction studies (Fig. 4C). Transduction with AAV2 and AAV2-AM was reduced by 2 to 3 orders of magnitude in the mAAVR knockout lines. However, while hAAVR supplementation restored AAV2 transduction to levels observed in wildtype cells, the restoration in AAV2-AM transduced mouse cells was much lower. Moreover, in human Huh7 cells AAV2 transduction was much higher than AAV2-AM consistent with the idea that AAV2-AM favors mAAVR over hAAVR (Fig. 4D).

### Supplementation with mAAVR and hAAVR showed differing effects on boosting AAV-LK03 transduction in vivo

**Figure 5.**
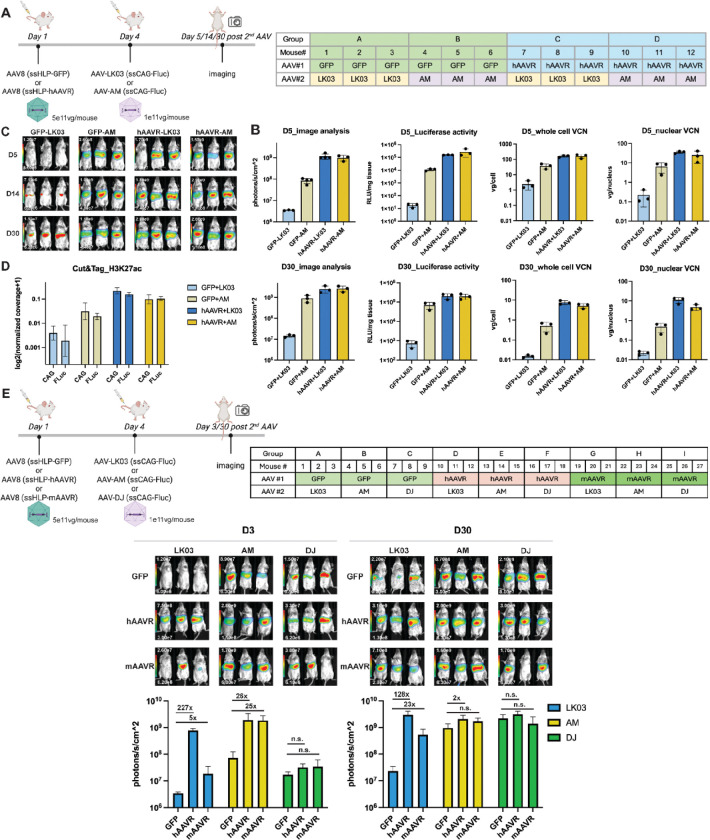
Supplementation of mAAVR and hAAVR showed distinct effects on boosting AAV-LK03 mediated transgene expression *in vivo*. (A) Experimental scheme is shown. BALB/c scid female mice received two batches of AAV injections. The first injection delivered 5e11vg per mouse with an AAV8 GFP or hAAVR expression cassette on day 1, and the second injection delivered 1e11vg per mouse AAV-LK03 or AAV-AM expressing the FLuc transgene on day 4. Mice were imaged on days 5, 14 and 30 post-second AAV injection and livers were collected on day 5 and 30 for analysis. (B) Analysis of images, luciferase activity and VCN from whole cell lysis or nuclear extracts. (C) Images taken on day 5, 14 and 30. (D) Cut&Tag analysis was done with day 30 samples against H3K27ac marks. The data for the marks covering the promoter region (CAG) and transgene (Fluc) are shown. (E) Experimental scheme. BALB/c scid female mice received two batches of AAV injections. The first injection delivered 5e11vg per mouse of an AAV8 expressing GFP, mAAVR or hAAVR on day 1, and the second injection delivered 1e11vg per mouse AAV-LK03 or AM or DJ expressing FLuc transgene on day 4. Mice were imaged on day 3 and 30 post second AAV injection and livers were collected on day 30 for analysis.

We tested hAAVR supplementation *in vivo* (Fig. 5A–D and Figure S5A-C). Two sequential AAV injections were performed in immunodeficient mice, with the first injection of an AAV8 expressing hAAVR or GFP (control) and the second injection of an AAV-LK03 or AAV-AM vector expressing a luciferase expression cassette. Mice were then imaged on days 5, 14 and 30 post the second AAV injection. hAAVR supplementation resulted in 47x more copies of hAAVR transcripts vs endogenous mAAVR transcripts at 30 days (Figure S5A). However, the exact difference in mouse vs human protein levels was not determined.

hAAVR supplementation enhanced AAV-LK03 and AAV-AM transduction by ~ 500 and 10x in mouse liver, respectively bringing AAV-LK03 and AAV-AM mediated transgene expression to similar levels (Fig. 5B–C and Figure S5B). The whole cell and nuclear vector copy numbers were determined at day 5 and day 30. At day 5, in all groups there was more whole cell vs nuclear vector copy numbers, as not all the vectors had not transversed into the nucleus. However, by day 30 most of the vector DNA was localized in the nuclear fraction (Fig. 5B). At day 5, AAV-LK03 and AAV-AM control animals (injected with AAV-GFP not AAV-hAAVR) showed ~ 10 times difference in vector DNA uptake that cannot explain the 500–1000x difference in luciferase expression, which was consistent with our previous studies^6^. The trend was similar when we compared an earlier time point, day 3 and day 30 (Figure S6). Transgene luciferase expression was 241 and 355-fold higher in AAV-AM vs AAV-LK03 treated animals at days 3 and 30, respectively. In general, transgene expression increased by ~ 10x between days 3 and 30 in each respective group while the VCN genomes decreased between days 3 and 30 in both groups. The relative drop in genome copies in AAV-LK03 was ~ 15x greater than the AAV-AM treated animals. However as mentioned (Fig. 5B), hAAVR supplementation increased VCN for both whole cell and nuclear lysates, indicating AAVR enhanced AAV trafficking resulting in more stable nuclear genomes at days 5 and 30 post vector infusion. At this time, we do not know if the hAAVR mediated increase in tissue and nuclear vector genomes results from greater vector uptake, internalization or vector stabilization.

In previous studies^6^, we showed that permissive but not repressive histone marks were more concentrated on vector genomes delivered by AAV-AM vs AAV-LK03 in mouse liver. As a preliminary study, we examined the permissive H3K27ac histone epigenetic mark at day 30 in liver nuclei after delivering AAV-LK03 or AAV-AM vectors with or without prior hAAVR supplementation (Fig. 5D). The results clearly demonstrate that hAAVR enhanced vector DNA H3K27ac concentrations consistent with enhanced luciferase mRNA and activity.

We then compared how mAAVR and hAAVR supplementation *in vivo* affected AAV-LK03, AAV-AM and AAV-DJ transduction (Fig. 5E, S5D). We first injected an AAV-8 vector expressing either mAAVR, hAAVR or GFP control and 3 days later an AAV-LK03, AAV-AM or AAV-DJ vector expressing a luciferase expression cassette in immunodeficient mice and then determined luciferase expression on days 3 and 30 post 2nd vector injection. Successful mAAVR supplementation was validated by ~ 100x increase in mAAVR transcripts on day 30 (Figure S5D). On day 3, hAAVR supplementation enhanced AAV-LK03-mediated luciferase expression by ~ 227-fold compared to the control (AAV8-GFP) mice, while mAAVR supplementation increased AAV-LK03-mediated luciferase expression by only 5-fold (Fig. 5E). In comparison, hAAVR and mAAVR supplementation equally boosted AAV-AM mediated transduction by 25-fold compared to AAV-GFP control animals. In contrast, mAAVR or hAAVR supplementation showed little to no enhancement in AAV-DJ mediated transduction suggesting that transduction by this capsid was less dependent on high levels of AAVR.

As expected, AAV-LK03, AAV-AM and AAV-DJ mediated transduction increased by day 30 regardless of AAVR supplementation. Nonetheless, the relative transduction of mAAVR supplemented AAV-LK03 group was still 6 times lower than the hAAVR supplemented LK03 group.

### Species-specific PKD2 sequences affect LK03 transduction.

To elucidate how species-specific AAVR affects species tropism of AAV vectors, we compared the protein sequence alignment of AAVR between mouse, human and non-human primates (NHP) (Figure S7, 6A). hAAVR shared 98.76% sequence identity with NHP AAVR, while there is only 84% identity shared by hAAVR and mAAVR.

**Figure 6.**
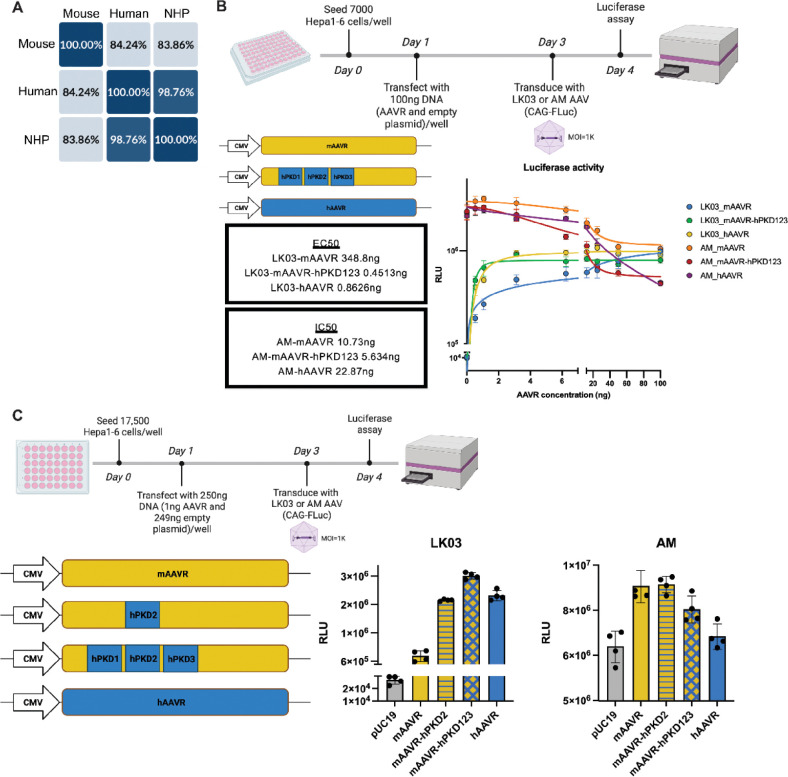
Species-specific PKD2 sequences affect LK03 transduction. (A) Sequence similarity derived from alignment of AAVR gene sequences from mouse, human and non-human primates. (B) Experimental scheme is shown. Luciferase activities were measured in Hepa1–6 cells transduced by AAV-LK03 or AAV-AM with varied levels of AAVR supplementation. EC50 and IC50 values were calculated by fitting curves through four-parameter non-linear regression. (C) Scheme of the experiment. Hepa1–6 cells were transfected with plasmids expressing mAAVR or hAAVR or indicated sequence-swapped AAVR variants, then transduced with AAV-LK03 or AAV-AM expressing the FLuc transgene and measured for luciferase activity.

To identify the key species-specific region of AAVR, we then performed sequence swap experiments between the variable portions of the mAAVR and hAAVR proteins. The PKD123 domain is a 283 amino acid stretch (Figure S7) with 19 amino acid differences between mouse and human AAVR. We substituted the hPKD123 sequence into the mAAVR sequence and constructed expression plasmids. Considering ligand-receptor binding is a dose-dependent phenomena, we transfected various amounts of the variant AAVR expression plasmids into Hepa1–6 mouse cells. The EC50 values were calculated (Fig. 6B) and showed that hAAVR and mAAVR-hPKD123 provided similar enhancement with AAV-LK03 mediated transduction, while mAAVR was much less efficient in enhancing AAV-LK03 transduction. Thus, the hPKD123 swap into the mAAVR was able to fully reconstitute hAAVR functionality (Fig. 6B). Consistent with the previous observations (Fig. 3, S3), AAV-AM transduction was more greatly enhanced by mAAVR vs hAAVR. We next studied the mAAVR-hPKD2, a 90 amino acid region (Fig. 6C) and found substitution of the hPKD2 sequences into mAAVR was sufficient to rescue AAV-LK03 transduction in mouse cells.

**Figure 7.**
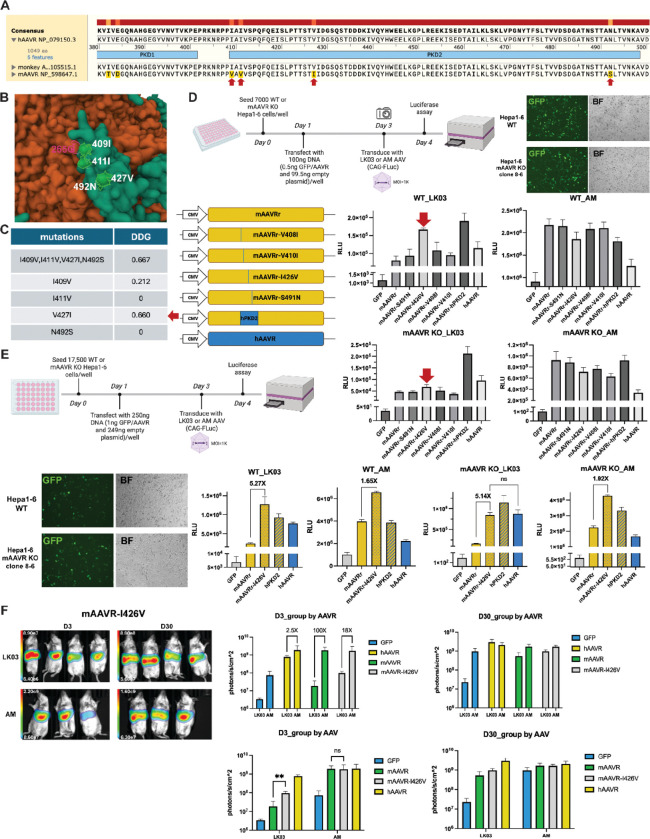
I426V variation within PKD2 across species is vital to LK03 transduction. (A) Alignment of AAVR sequences across species, revealing the four amino acid variants within the PKD2 region. (B) Cyro-EM structure of AAV2-hAAVR complex with zoom into the 265G site of the capsid and four amino acids from the PKD2 region. PDB ID: 6IHB. (C) Prediction with SSIPe of binding affinity changes (ΔΔG_bind_) of AAV2-hAAVR complex with mutated amino acids as indicated. Arrow points out that V427I mutation showed the greatest change on binding affinity. (D, E) Experimental scheme is shown. Wildtype or mAAVR knockout Hepa1–6 cells were transfected with plasmids expressing mAAVR, hAAVR or the indicated sequence-swapped AAVR variants, then transduced with AAV-LK03 or AAV-AM expressing the FLuc transgene and measured for luciferase activity. The arrow points out that I426V of mAAVR variant showed the most impact on LK03 transduction. (F) BALB/c scid female mice received two sets of AAV injections. The first injection delivered 5e11vg per mouse AAV8 expressing the mAAVR-I426V transgene on day 1, and the second injection delivered 1e11vg per mouse AAV-LK03 or AAV-AM expressing the FLuc transgene on day 4. Mice were imaged on day 3 and 30 post second AAV injection.

Alignment with species-specific AAVR sequences identified four amino acids that are conserved in human and NHP but varied in mouse (Fig. 7A). Cryo-EM structure of the AAV2-hPKD2 complex showed that these four amino acids bind close to the 265G capsid residues (Fig. 7B). The SSIPe program is a method to calculate binding affinity changes (ΔΔG_bind_) of protein-protein interactions upon amino acid changes at the protein-protein interface^11^. This program predicted that changing the four amino acids from hPKD2 to mPKD2 was unfavorable for its binding to AAV2 (Fig. 7C). Among them, the V427I substitution was predicted to have the largest impact. We then examined how these four amino acids individually affected AAV-LK03 and AAV-AM transduction by comparing mAAVR variants with swapped single amino acids in the PKD2 region (Fig. 7D–E). The mAAVR-I426V variant resulted in the greatest increase in AAV-LK03 transduction in both wildtype and mAAVR knockout Hepa1–6 cells.

We further investigated the effects of mAAVR-I426V variant *in vivo* (Fig. 7F). Added mAAVR-I426V enhanced AAV-LK03 transduction much more than exogenously added mAAVR. The difference in AAV-LK03 vs AAV-AM transduction was 100-fold with supplemented mAAVR but only 18-fold when the mAAVR-I426V variant was supplied. Thus, the single amino acid substitution in the mAAVR-I426V variant provided substantial but not fully active hAAVR activity in mouse liver.

We also quantified the binding activity of species-specific PKD2 peptides with the LK03 or AM AAV capsids by an enzyme-linked immune sorbent assay (ELISA) (Figure S8). Mouse PKD2 (mPKD2), human PKD2 (hPKD2) and mouse PKD2 containing the I426V substitution (mPKD2-I426V) were cloned into CMV driving vectors with six-histidine tags and transfected into HEK293 cells (Figure S8A). PKD2 peptides were purified with Nickel charged agarose and validated by coomassie blue staining (Figure S8B). Next, an ELISA assay was performed to measure the binding avidity of PKD2 peptides with AAV-LK03 or AM. As shown in Figure S8B, AAV-LK03 showed the lowest binding with mPKD2 and highest binding with hPKD2, while AM showed the best binding with mPKD2. This result was consistent with our functional findings that LK03 and AM have differential preferences for AAVR utilization.

### hAAVR supplement protects AAV-LK03 from degradation and enhances episome formation.

**Figure 8.**
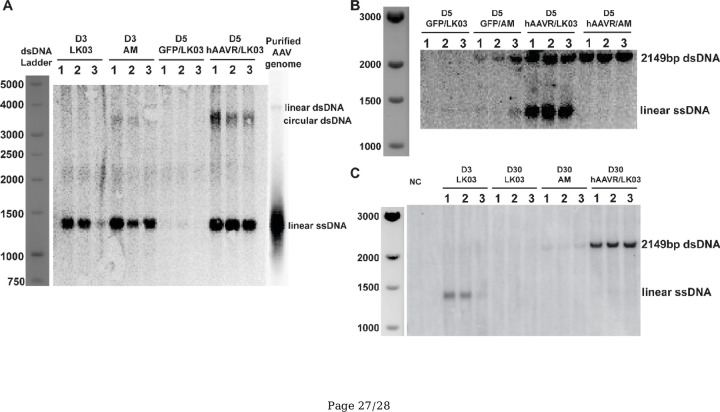
hAAVR supplement protects AAV-LK03 from degradation and enhances episome formation. (A-C) Southern blotting with liver samples as indicated. (A) Samples were digested with a vector no-cutter enzyme Xho I overnight before loaded to agarose gel. (B, C) Samples, including the negative control (NC) was DNA extracted from an uninjected mouse liver. DNA samples were digested with a vector two-cutter enzyme Bbs I-HF overnight before loaded to agarose gel. After DNA transfer to the membrane the blots were hybridized with a [<−^32^P] dCTP that will recognize the AAV-luciferase vector genomes. In A, circular monomeric and single-stranded genomes were detected. In B and C, the 2149bp band represents all double-stranded luciferase vector DNA..

To determine the molecular structures of the vector genomes, we performed Southern blotting (Fig. 8). At day 3, most of the AAV-LK03 and AAV-AM delivered genomes were present as single-stranded genomes (Fig. 8A, C). By day 5, without AAVR supplementation, most of the vector genomes were present as dsDNA. However, at day 5, with prior hAAVR supplementation versus controls, the number of dsDNA AAV genomes was higher and comparable in both AAV-AM and AAV-LK03 treated animals. This was consistent with the enhanced luciferase expression observed in hAAVR supplemented animals (Fig. 5B). Of interest was the finding that number of ssDNA vector genomes with AAV-LK03 was also increased at day 5 with hAAVR supplementation and barely detectable by day 30 (Fig. 8C). The reason for this is unclear at the present time.

## DISCUSSION

Species tropism of AAV vectors historically hinders translation from pre-clinical characterization of AAV in rodent models to primate subjects. Since the discovery that KIAA0319L or AAVR was an essential receptor to AAV transduction^7^, a number of studies have been reported on the structural analysis of the AAV-AAVR interaction^12–18^ or AAVR engineering for optimizing AAV transduction^19–21^. While the Chapman group compared the binding avidity of hAAVR with goat AAV and AAV2 or AAV5^22^, very little information regarding AAVR differences from the species commonly used in preclinical evaluation of AAV vectors is available. Our study exemplifies the importance of how small differences in the AAVR sequence based on species variation affects its association and transduction efficiency with various AAV capsids. In addition to our studies here comparing AAV-LK03 and AAV-AM containing a single amino acid variant, there are other recently described capsids of potential interest for further study. For instance, the Vandenberghe group, using barcoded AAV libraries in mice and nonhuman primates, identified a single amino acid variation in capsids, which alters liver tropism of vectors across species^23^. The Asokan lab found that swap of VP1/2u region between AAV8 and Avian AAV rescued low transduction of Avian AAV in human cells^24^. The Lisowski group demonstrated a threonine insertion at position 265 within variable region I as a key residue conferring murine hepatic transduction to human-derived clade B and C variants^1^. Unraveling the role of species specific AAVR with these and similar vectors may provide new insights into AAV transduction mechanisms.

AAV-LK03 has been used in clinical studies^5^ but required surrogate capsids for rodent efficacy studies because of its inability to effectively transduce rodent cells *in vitro* or *in vivo*. In our study, we demonstrate that AAVR is an important host factor that results in the discordance across species, exemplified by AAV-LK03. Other chimeric capsids isolated from our lab including AAV-NP59 and NP-40^25^ have similar discordance in their inability to transduce murine cells. This vector contains the same sequence around 265G as its AAV2 parental serotype and further studies are required to determine if species specific AAVR is involved.

Only a single glycine insertion at site 265 was required to change the tropism of AAV-LK03 that changed the tropism to include both murine and primate cells. Here, by capsid sequence alignment and investigating Cryo-EM structures of multiple AAV serotypes, we showed that 265G was located in a surface-exposed region of the virion, allowing direct interaction of AAV particles with host factors including AAVR. In fact, sequence swap between mAAVR and hAAVR confirmed that four amino acid variation within PKD2 domain was vital to LK03 transduction, I426V of which showed the most impact. Therefore, based on the structural and functional studies shown in our study, the AAVR PKD2 domain and the capsid 265–270 amino acid region appear to be a vital interacting region.

Interestingly, hAAVR supplementation resulted in a slight reduction in AAV-AM mediated transduction in mouse cells. This is consistent with a possible AAV-AM preference for mAAVR over hAAVR. Taken together, these data suggest that differential AAVR-Capsid interactions are important factors in determining the relative tropism in vector transduction.

A remaining major question is the mechanism behind differential AAV-capsid-AAVR binding having such a large effect on AAV-mediated transgene expression. The process of AAV transport from the cell membrane into the nucleus where uncoating occurs is complex with AAVR perhaps playing multiple roles in the pathway^26^. In our culture cell data, hAAVR expression did not have a large effect on nuclear DNA copy number but ~ 100x increase in transgene expression. In mouse liver, hAAVR expression did affect cellular episome copy number but cannot entirely explain the much greater level of transgene expression. It is possible that AAVR differentially alters the capsid structure that then has an effect on nuclear capsid uncoating and/or genome stability. We have previously found that AAV-LK03 and AAV-AM is highly discordant in transgene expression in mouse liver and expression correlates with differential permissive histone marks^6^. However, over time the number of AAV-LK03 genomes are more greatly reduced compared to AAV-AM. In this study, we did show that hAAVR expression in mouse liver also enhanced the concentration of a permissive histone mark on AAV delivered genomes. While this study clearly demonstrates the importance of specific variable sequence differences in AAVR between species in transduction efficiencies, more work is required to understand the mechanism for this effect. Unraveling these mechanisms may provide better capsid designs and perhaps offer approaches to better predict dose-responses between individuals receiving rAAV vectors.

## MATERIALS & METHODS

### Cell culture

Huh7 (JCRB, Cat #JCRB0403), HEK293T (ATCC, Cat #CRL-3216), Hepa1–6 (ATCC, Cat #CRL-1830), Hela (ATCC, Cat #CRM-CCL-2), NIH3T3 (ATCC, Cat #CRL-1658), C1C12 (ATCC, Cat #CRL-1772), A549 (ATCC, Cat #CCL-185), MCF7 (ATCC, Cat #HTB-22) cells were cultured with DMEM medium with 10% fetal bovine serum (FBS), 2mM L-glutamine, 2mM sodium pyruvate, and 1% antimycotic-antibiotic in a humidified incubator at 37°C with 5%CO_2_. Expi293F cells (Thermo Fisher #A14527) were cultured in Expi293 Expression medium (Thermo Fisher #A1435101) in a humidified shaker incubator at 37°C following product instructions.

### Animal procedures

Mouse experiments and procedures were approved by the Institutional Animal Care and Use Committee (IACUC) at Stanford University. Six- to eight-week-old white BALB/c scid female mice (Stock #001803) were purchased from the Jackson Laboratory.

rAAV was delivered by tail vain injections with indicated doses diluted to 150μL volume per mouse. At desired time points, in vivo firefly luciferase imaging was performed by intraperitoneal injection of 150 μg D-Luciferin (Biosynth #L-8220) per g body weight. Five min after Luciferin injection, mice were imaged for ventral luciferase readings using an Ami imaging system.

At the end of each experiment, mice were anesthetized with isoflurane for liver harvesting.. Liver tissue was quickly cut into pieces. Tissues for mRNA analysis were immediately immersed in RNAlater solution (Thermo Fisher #AM7020), stored at 4°C overnight and transferred to −20°C for long-term storage. Tissues for luciferase activity analysis and vector genome copy quantification were flash frozen on dry ice and stored at −80°C.

### Plasmid construction

Plasmids to package AAVs that were used in most experiments of this study can be accessed through Addgene: pAAV-CAG-FLuc (#83281), AAV-LK03 (#206512), AAV-AM (#206513), pAAV-CAG-RLuc (#83282), pAAV-CAG-GFP (#37825), pAAV-CAG-TdTomato (#59462). PB-CRISPR plasmid that was used to generate mAAVR knockout cells was from Addgene (#160047)^27^. Empty vector pUC19 can be accessed from Addgene (#50005).

Vectors expressing capsid protein were generated with In-Fusion Snap Assembly (Takara #638947) by cloning amplified capsid fragments into a plasmid backbone driven by CMV promoter. Vectors expressing AAVR variants with swapped sequences as well as PKD2 peptides were generated in the same manner.

For AAVR variants with single amino acid mutations, Q5 High-Fidelity 2X mastermix (NEB #M0492S) was used for PCR reactions using pCMV-mAAVR as template. Reactions were digested with Dpn I for 1h and purified with DNA clean & concentrator kit (Zymo Research #D4013). Purified reactions were then transformed into Stellar Competent cells (Takara #636763). Colonies were picked for sequencing to validate point mutations.

GFP- or AAVR-expressing vectors driven by the HLP promoter were generated with In-Fusion Snap Assembly by cloning the transgenes into a plasmid backbone (Addgene #109313).

### Production of AAV

Recombinant AAV vectors were produced with triple transfection either in HEK293T or in Expi293F cells as previously described^6^. Briefly, for HEK293T transfection, 8E6 cells were seeded per 15cm plate in 25mL media on day 1. On day 2, transfection mastermix containing 3.75μg pRep-Cap, 3.75μg Gene of interest (GOI), 12μg pAd5 plasmids and 105μL PEI in 2mL Opti-MEM was mixed well by pipetting and incubated for 20 min at room temperature. 2mL of mastermix was added directly into the plates. After a 72h incubation, cells were dissociated by adding 1/80 volume of 0.5M EDTA (pH8.0) to the medium and pelleted by centrifuge. AAV was purified with AAVpro Purification kit Maxi-All serotypes (Takara #6666) following the manufacturer’s instruction. Usually, 10–20 plates of cells can be purified with 1 column.

For Expi293F transfection, cells were seeded with a density of around 1E6 cells/mL on day 1. On day 2, cells were transfected by adding 10% volume of mastermix with ratios of 0.75μg DNA/1E6 cells and 1.5mL FectoVIR (Polyplus-transfection #120 – 100)/1mg DNA. Ratio of the pHelper: Rep/Cap: GOI plasmids is 0.43:0.36:0.22. Mastermix was mixed and returned to the shaker incubator for 30min. After pipetting up and down, the drops were added to the cells. After a 72h incubation, cells were harvested and AAV was purified with AAVpro Purification kit Maxi-All serotypes (Takara #6666). Purified AAV was aliquoted at −80°C until further use.

For AAV titration, AAV genome was extracted with QIAamp MinElute Virus Spin kit (Qiagen #57704) and tittered by qPCR with serial dilutions of linearized GOI plasmid as standard. Apex qPCR Green Master Mix (Genesee Scientific #42–116PG) was used for qPCR reactions following the manufacturer’s instruction.

### Cell transfection and transduction

Cells were transfected with Lipofectamine 3000 Transfection reagent (Thermo Fisher #L3000015) following the manufacturer’s instruction.

For AAV transduction, culture medium was changed with Opti-MEM containing AAV diluted to the indicated MOI. 6h later, the media was changed back to full cell culture media.

### Luciferase assay

ONE-Glo luciferase assay system (Promega #E6110) was used for testing luciferase assay for most experiments. With cultured cells, wells were washed with PBS first and then 1:4 PBS-diluted ONE-Glo substrate was added directly to the wells, and gently rocked for 10min. Plate reading was performed with a Veritas luminometer system using GloMax software. For frozen liver tissues, tissue weight was measured and recorded. Tissue was homogenized in RINO 1.5-mL Screw-Cap tubes filled with stainless steel beads in 200μL 1x Passive Lysis buffer (Promega #E1941) using homogenizer (Next Advance Bullet Blender Storm–BBY24M) at speed 8 for 3min at 4°C. Liver extract was collected from supernatant after centrifuge at 9600g for 10min at 4°C. 5μL liver extract was added to 100μL ONE-Glo substrate, incubated for 10min with gentle rocking and measured in plate reader. Measured luciferase activity was normalized to tissue weight.

Dual luciferase assay was performed with Dual-Glo luciferase assay system (Promega #E2920) following the manufacturer’s instruction.

### RNA extraction, cDNA synthesis and RT-qPCR

With TRI-reagent (Zymo Research #R2050–1-50) directly added to wells of cultured cells or mixed with liver tissues in a homogenizer, RNA was then extracted and purified with Direct-zol RNA miniprep kit (Zymo Research #R2071). cDNA was prepared with Maxima First Strand cDNA Synthesis kit (Thermo Fisher #K1641). RT-qPCR was using Apex qPCR Green Master Mix (Genesee Scientific #42–116PG) following manufacturer’s instruction.

### Vector copy number (VCN) quantification

To quantify the vector copy number from whole cell lysates, cell pellets were harvested from culture plates or liver extracts described above from homogenized liver tissues were extracted with Quick DNA Miniprep kit (Zymo Research #D3024) for DNA including genomic DNA from both host cells and AAVs.

To quantify the vector copy number from nuclear extracts, nuclei from cultured cells were isolated with NE-PER Nuclear and Cytoplasmic Extraction Reagents (Thermo Fisher #78833). Nuclei from liver tissues were isolated following the manual of CUTANA CUT&Tag kit (EpiCypher #14–1102).

Purified genomic DNA was titered by qPCR using series dilutions of linearized AAV GOI plasmid and mouse genomic DNA (Promega #G3091) as standards.

### ELISA assay

HEK293 cells were transfected with PKD2-expressing vectors with a six-Histidine tag and recombinant PKD2 peptides were purified with a His-Spin protein miniprep kit (Zymo Research #P2001) and validated by Coomassie Blue staining. ELISA assays were performed as previously described^22^. Wells of 96-well plate were coated with 50μL/well 2.5μg/mL AAV-LK03 or AM overnight, followed by blocking with 5% milk-containing TBST and incubation with PKD2 peptides as indicated. Wells were then washed and incubated with anti-His-HRP antibody (R&D Systems #MAB050H-SP; 1:1500 dilution), followed by ultra-TMB substrate addition. Plates were read for absorbance at 450nm in a BioTek microplate reader. EC50 values were calculated by fitting curves through five-parameter non-linear regression.

### Southern blotting

Liver samples were extracted for DNA with DNeasy Blood & Tissue kit (Qiagen). To acquire a DNA of high concentration and good quality, 75 μL elution buffer that was pre-warmed to 70°C was directly added to column, followed by incubation at 70°C for 20min. 10 μg DNA for each sample was digested at 37°C overnight either with Xho I that cuts host genomic DNA but not the AAV genome, or with Bbs I-HF that cuts AAV genome at two sites and generates the 2149bp band. Purified ssDNA genome from 10^7^ vg AAV-LK03 (CAG-Fluc) was boiled at 95°C for 5min in the presence of 50% Formamide and snap cooled on ice. Digested DNA was loaded to an ethidium bromide-containing 1% agarose gel (10X6 inch) and run at 40V overnight (~ 19h). The gel was imaged with a ChemDoc instrument (Bio-Rad). The gel was washed with denaturing buffer (3M NaCl and 400mM NaOH) for 10min with agitation. The DNA was transferred to Hybond N + Nylon membrane (Fisher) using transfer buffer (3M NaCl and 8mM NaOH) overnight. The membrane was washed with 2X saline sodium citrate (SSC) buffer for 5min with agitation to remove transfer buffer residue. After crosslinking, the membrane was blocked with QuikHyb hybridization solution containing 10 μg/mL UltraPure Salmon Sperm DNA at 65°C for 1h. For probe synthesis, the 489bp amplicon corresponding to the FLuc sequence was gel purified from PCR products derived from the plasmid AAV-CAG-FLuc as a template and primers (Forward: GCTGGTGCCCACACTATTTA; Reverse: ACCTGGTAGCCCTTGTATTTG). A 200ng amplicon was labeled with [<−^32^P] dCTP using the BcaBEST Labeling Kit (Takara) according to manufacturer’s instructions. Unincorporated nucleotides were removed with an Illustra Microspin G-25 column. The probe was added to the pre-hybridized membrane and incubated at 65°C with rotation overnight. The membrane was washed with 2X SSC buffer and 2X SSC containing 0.1% SDS at 65°C for 10min twice. The membrane was exposed onto a phosphoimager screen for optimized time, visualized in a PharosFX Molecular Imager (Bio-Rad) and analyzed with Quantity One 1-D software v4.6.8 (Bio-Rad).

### Re-analysis of published RNAseq data

Raw RNA sequencing data were obtained from NCBI Sequence Read Archive (SRA) under ERP013191^10^.

For data preprocessing, all raw sequencing FASTQ files were downloaded with SRA-Toolkit (v 3.0.3) and processed with fastp^28^ (v0.24.3) to remove adaptor sequences, duplication and low-quality sequences. For single-end data, the parameters used are -i and -o for input and output files, --dedup. For paired-end data, the parameters used are: -i, -I for input R1 and R2; -o, -O for output R1 and R2; -- dedup. Reference sequences were obtained from the NCBI Reference Sequence Database (https://www.ncbi.nlm.nih.gov/refseq/). For human, we used version GCF_000001405.40_GRCh38.p14 as reference genome and transcriptome. For mouse, we used GCF_000001635.27_GRCm39 for both reference genome and transcriptome.

For data analysis, preprocessed FASTQ files are then quantified using Salmon^29^ (v1.10.3). We construct the “decoy-aware” transcriptome using the entire genome as “the decoy sequence”. First, we extract the decoy list from the reference genome, followed by concatenating reference transcriptome and genome to one comprehensive file, and then construct the decoy-aware transcriptome index using salmon index with default parameters as described in https://combine-lab.github.io/alevin-tutorial/2019/selective-alignment/. We then quantify each of the sequencing samples using the following command: For single-end samples: salmon quant -i (corresponding salmon index) -l SR -r (cleaned files from the preprocessing step) -o (the output folder)

For paired-end samples: salmon quant -i (salmon index prepared above) -l ISR – 1 (cleaned R1) −2 (cleaned R2) -o (the output folder)

Therefore, for each sample, we get quantifications of each of the transcripts including their isoform expressions in the output file “quant.sf”.

### Cut&Tag

Day 30 liver samples were processed as previously described^6^. Reagents and protocol are commercially available by Epicypher (https://www.epicypher.com/resources/protocols/cutana-cut-and-tag-protocol/). Briefly, for nuclei harvest, frozen liver samples were homogenized with RINO 1.5-mL Screw-Cap tubes filled with stainless steel beads in NE buffer and incubated in NE buffer on ice for 20min. 1e5 nuclei per sample were mixed with activated Concanavalin A beads, followed by overnight incubation with primary antibody (Thermo Fisher) targeting H3K27ac marks and 30min incubation with secondary antibody (Epicypher). After wash, CUTANA pAG-Tn5 was added and incubated for 1h. Tagmentation was performed for 1h, followed by a wash with TAPS buffer. DNA was released by adding SDS release buffer and incubation at 58°C for 1h. Samples were then quenched by SDS quench buffer. The beads were removed with magnets and PCR was performed with the DNA for 21 cycles. The amplified library was cleaned up with AMPure beads, eluted in 17uL 0.1x TE buffer. A bioanalyzer was used to verify library qualities before pooling samples for sequencing. Sequencing was performed on NovaSeq X Plus Series (PE150) generating ~ 13.8M paired-end reads per sample.

### Data preprocessing

Reads were first trimmed with classical Illumina adapters using Trimmomatic^30^ (v0.40).

Mouse reference genome GRCm39 assembly GCF_000001635.27 was obtained from the NCBI Reference Sequence Database^31^ (https://www.ncbi.nlm.nih.gov/refseq/) and then merged with viral genome sequence (gAAV). The gAAV is the same as plasmid pAAV_CAG_FLuc from 5’ ITR to 3’ ITR.

### Data alignment and downstream analysis

We then used Bowtie2^32^ (v2.5.2) to build index with the above merged reference and align our processed reads with the following parameters^6^: –local–very-sensitive-local–nounal–no-mixed–no-discordant–phred33 -I 10 -X 700. The resulting bam files were then sorted and indexed with Samtools^33^ (v1.19.2). Duplicates were marked and removed using Picard^34^ (v3.2.0) with parameters VALIDATION_STRINGENCY = LENIENT ASSUME_SORTED = true REMOVE_DUPLICATES = true, then indexed using Samtools.

We calculated coverage at each position from the above processed bam files using deeptools^35^ (v3.5.4) --outFileFormat bedgraph --binsize 1 --normalizedUsing CPM and then calculated the mean coverage value of each region as $\frac{\sum_{\mathrm{region\_start}}^{\mathrm{region\_end}}\;\mathrm{normalized\_count}}{\mathrm{region\_size}}$.

### Statistics

Statistical analysis was performed with GraphPad Prism 10 (Version 10.2.2).

## Supplementary Material

Supplementary Files

This is a list of supplementary files associated with this preprint. Click to download.

• blotimages.pdf

• Supplementalfigures.pdf

## Data Availability

Data from PDB database analyzed in this study can be accessed through https://www.rcsb.org. PDB ID are listed as below: 6IH9 (AAV2); 8A9U (AAV3b); 6JCR (AAV1); 6PWA (AAV8); 7WJW (AAV9); 3J1Q (AAV-DJ); 6IHB (AAV2-hAAVR). RNAseq data re-analyzed in this work can be accessed in Sequence Read Archive (SRA) with SRA# ERP013191.
